# H5N1 avian influenza re-emergence of Lake Qinghai: phylogenetic and antigenic analyses of the newly isolated viruses and roles of migratory birds in virus circulation

**DOI:** 10.1099/vir.0.83419-0

**Published:** 2008-03

**Authors:** Guihua Wang, Dawei Zhan, Laixing Li, Fumin Lei, Bohua Liu, Di Liu, Haixia Xiao, Youjun Feng, Jing Li, Baoan Yang, Zuohua Yin, Xiaohui Song, Xiaojia Zhu, Yanlong Cong, Juan Pu, Jian Wang, Jinhua Liu, George F. Gao, Qingyu Zhu

**Affiliations:** 1 Center for Molecular Virology and Center for Molecular Immunology, Institute of Microbiology, Chinese Academy of Sciences, Beijing 100101, PR China; 2 Graduate University, Chinese Academy of Sciences, Beijing 100049, PR China; 3 China-Japan Joint Laboratory of Molecular Immunology and Molecular Microbiology, Institute of Microbiology, Chinese Academy of Sciences, Beijing 100080, PR China; 4 State Key Laboratory of Pathogens and Biosecurity, Academy of Military Medical Sciences, Beijing 100071, PR China; 5 Institute of Microbiology and Epidemiology, Academy of Military Medical Sciences, Beijing 100071, PR China; 6 Northwest Institute of Plateau Biology, Chinese Academy of Sciences, Xi'ning 810008, PR China; 7 Institute of Zoology, Chinese Academy of Sciences, Beijing 100080, PR China; 8 College of Veterinary Medicine, China Agricultural University, Beijing 100094, PR China; 9 Beijing Genomics Institute, Chinese Academy of Sciences, Beijing 101300, PR China

## Abstract

Highly pathogenic avian influenza H5N1 virus has swept west across the globe and caused serious debates on the roles of migratory birds in virus circulation since the first large-scale outbreak in migratory birds of Lake Qinghai, 2005. In May 2006, another outbreak struck Lake Qinghai and six novel strains were isolated. To elucidate these QH06 viruses, the six isolates were subjected to whole-genome sequencing. Phylogenetic analyses show that QH06 viruses are derived from the lineages of Lake Qinghai, 2005. Five of the six novel isolates are adjacent to the strain A/Cygnus olor/Croatia/1/05, and the last one is related to the strain A/duck/Novosibirsk/02/05, an isolate of the flyway. Antigenic analyses suggest that QH06 and QH05 viruses are similar to each other. These findings implicate that QH06 viruses of Lake Qinghai may travel back via migratory birds, though not ruling out the possibility of local circulation of viruses of Lake Qinghai.

Highly pathogenic avian influenza (HPAI) H5N1 virus emerging as an infectious entity has raised great concerns on public health globally, since the first human fatal cases reported in Hong Kong, China 1997 (Claas *et al.*, 1998; Hien *et al.*, 2004; Subbarao *et al.*, 1998). From 1997 onwards, H5N1 avian influenza virus (AIV) has existed as a serious threat to human health worldwide. As of August 2007, a total of 322 human cases of H5N1 infection has been recorded, including 195 cases of death (WHO, 2007). Undoubtedly, H5N1 possesses a serious threat to public health as well as to the global economy, so preparedness for such a threat is a global priority (Liu *et al.*, 2005; Webster & Hulse, 2005; WHO, 2005).

Influenza A virus is a negative-sense, single-stranded RNA virus. It has eight gene segments coding for 11 proteins, in which two integral surface glycoproteins, haemagglutinin (HA) and neuraminidase (NA) harbour 16 and 9 serotypes, respectively, resulting in multifarious subtypes with different combinations (e.g. H1N1, H3N2, H5N1) (Chen *et al.*, 2001; Hinshaw *et al.*, 1980; Palese, 1977; Webster *et al.*, 1992). To date, diverse animals, including domestic birds, wild birds and mammals, are reported to be infected by H5N1, suggesting that H5N1 might overcome the interspecies barriers (Chen *et al.*, 2005; Crawford *et al.*, 2005; Enserink & Kaiser, 2004; Guan *et al.*, 2000; Keawcharoen *et al.*, 2004; Kuiken *et al.*, 2006; Liu *et al.*, 2005; Smith *et al.*, 2006a). In general, all subtypes persist in evolutionary equilibrium (*evolutionary stasis*) and seldom show clinical signs in their natural hosts, the wild waterfowl (Guan *et al.*, 2000; Webster *et al.*, 1992). However, it was unexpectedly observed that H5N1 viruses caused the sporadic death of wild migratory birds in Hong Kong, 2002 and these birds were demonstrated extremely pathogenic to ducks by further animal experiments (Sturm-Ramirez *et al.*, 2004). It was the first report of the fatal cases in wild aquatic birds caused by AIV since 1961 (Becker, 1966).

Nevertheless, the H5N1 outbreak of Lake Qinghai, China, 2005, astonished the world, in that migratory birds were observed with the infection and over 6000 birds died (first H5N1 outbreak in wild bird population) (Chen *et al.*, 2005, 2006; Liu *et al.*, 2005). In view of Lake Qinghai's geographical status in bird migration, the role of migratory birds, possibly as the carrier in the circulation of the viruses along the flyway, has been debated extensively (Kilpatrick *et al.*, 2006; Normile, 2006; Poland *et al.*, 2007). It is believed that, through the overlapping flyways, the HPAI H5N1 viruses have become prevalent among different migratory bird species, and have allowed for the spread of the virus across continents. The epidemics that broke out in Europe and Africa have resulted in tremendous economic losses, presenting clues and evidence of the key role of migratory birds in H5N1 epidemiology.

One year after the QH05 outbreak, the fatal H5N1 viruses were re-emerging in some areas of the Qinghai Province and Tibet Autonomous Region, China, and caused more species of birds to become infected. In this study, we present the genetic and antigenic characteristics of the latest isolates and propose possible explanations for AIV re-emergence in Qinghai, proposing the potential roles of the migratory birds in the H5N1 AIV circulation.

To analyse the viral agents in the 2006 outbreak, a total of 87 specimens including oropharyngeal and cloacal swabs was collected from 12 dead birds of Lake Qinghai, Maduo County, Yushu County in Qinghai province, China during 6–15 May, 2006. The pre-treated samples were inoculated in 10-day-old embryonated specific-pathogen-free (SPF) eggs as described previously (Liu *et al.*, 2005), and isolates were identified and subtyped by both haemagglutinin inhibition (HI) and neuraminidase inhibition assays. In total, six H5N1 viruses were isolated and named as A/great black-headed gull/Qinghai/01/06 (A/GbhGull/QH/01/06), A/bar-headed goose/Qinghai/01/06 (A/BhGoose/QH/01/06), A/bar-headed goose/Qinghai/02/06 (A/BhGoose/QH/02/06), A/migratory bird/Qinghai/01/06 (A/Mbird/QH/01/06), A/bar-headed goose/Qinghai/11/06 (A/BhGoose/QH/11/06) and A/great black-headed gull/Qinghai/12/06 (A/GbhGull/QH/12/06), respectively.

To detect antigenic characteristics of the QH06 viruses, we compared cross-activity between QH06 virus and other H5N1 viruses by HI assay as described previously (Swayne *et al.*, 1998). The strains A/BhGoose/QH/01/06 and A/BhGoose/QH/1/05 were chosen as the representatives of QH06 and QH05 viruses, respectively, and strains A/Vietnam/1194/2004 and A/Beijing/01/2003 were used as experimental controls. Antisera to these viruses were prepared in mice. As shown in Table 1, mouse antiserum against A/Vietnam/1194/2004 cross-reacted with lower titres with the other three isolates from China. Similar results were also obtained when A/Vietnam/1194/2004 cross-reacted with the antisera of the other three isolates. These results suggest that QH06 viruses resemble the viruses A/BhGoose/QH/1/05 and A/Beijing/01/2003, but are antigenically differentiated from the A/Vietnam/1194/2004 virus.

QH06 viruses pathogenicity testing (intravenous pathogenicity index, IVPI) in chickens was performed in accordance to the recommendations of the OIE (2004). Briefly, groups of ten 6-week-old SPF-White Leghorn chickens (purchased from Beijing Merial Vital Laboratory Animal Technology Ltd), housed in negative-pressure isolator cages, were inoculated intravenously (i.v.) with 0.2 ml of a 1 : 10 dilution of bacteria-free allantoic fluid (10^8.45^ EID_50_ per 0.1 ml) to determine the IVPI. We observed that all inoculated chickens died within 24 h and showed that the highest intravenous pathogenicity was 3.0. Tissue analyses for gross lesions and histopathology of the dead chickens presented similar systemic lesions to those observed in chickens inoculated with QH05 viruses. The most consistent gross lesions included severe pulmonary oedema with congestion and haemorrhage, oedema of the brain, and petechial to ecchymotic haemorrhages in the skin, fat pads, and the caecal tonsil. Histologically, microscopic lesions comprised haemorrhage, oedema and necrosis in multiple visceral organs, and congestion, haemorrhage of the pulmonary and hepatic tissues. To test the pathogenicity in mammalian hosts, groups of eight 18–22 g female BALB/c mice (Beijing Laboratory Animal Research Center, Beijing, China) were infected intranasally (i.n.) with 50 μl 10^6^ EID_50_ per 0.1 ml under anaesthesia. The 50 % minimal lethal dose (MLD_50_) was determined by i.n. inoculation and calculated by the method of Reed & Muench (1938). The results showed that all six isolates were found to be lethal to mice. The infected mice began to show signs of illness 2 days post-infection, such as ruffled fur and hunched posture. One mouse from the group A/GbhGull/QH/01/06 died on the second day, while death started in other groups on the third day. All mice died on day 5 post-infection. The mean death time of the inoculated mice was 3.8 days. Necropsy examination revealed a haemorrhagic lung and a swollen liver. Histologically, microscopic lesions showed severe haemorrhagic and histiocytic pneumonia with the infiltration of neutrophils, including severe meningeal and parenchymal haemorrhage with an infiltration of lymphocytes in the brain, severe pancreatic necrosis and severe nephrosis. All results indicate that the QH06 viruses retain the properties of high pathogenicity to both chicken and mouse thereof being named as HPAI H5N1.

We further sequenced the genomes of the six QH06 isolates by using a method described previously (Liu *et al.*, 2005). We obtained all eight RNA sequences with complete coding sequences of each of the six QH06 isolates. These data were then aligned with sequences of the QH05 viruses and of the overseas viruses, along the migratory bird flyways, by using clustal_x (version 1.81) (Thompson *et al.*, 1997). The alignments show that all HA segments maintain the multiple basic amino acids cleavage site QGERRRKKR/G, a 20 aa deletion in the NA stalk (residues 49–68) and E627K substitution in PB2 (except for strain A/GbhGull/QH/12/06 which has an E627). From the comparison of the consensus sequences of QH05 and five QH06 viruses (except A/GbhGull/QH/12/06), we noted 7 aa variations, including N158D and V214I in HA, V99I in NA, K100R in NP, M483V in PB2 and G70V in PB1-F2. The inspected QH06 PB1-F2 proteins are all 90 aa long and capture a Val (V) at position 70, which is dominated by Gly (G) in QH05 with the exception of Glu (E) in A/BHGs/QH/2/05. Since PB1-F2 is recognized as a new virulence contributor and functions in reducing the immune responses as well as increasing the cytotoxicity via the induction of macrophage apoptosis (Chen et al., 2001; Coleman, 2007; Zamarin et al., 2005, 2006), this mutation might be of interest. Particularly, six of the seven sites in A/GbhGull/QH/12/06 are identical to QH05 viruses, with the exception that position 100 in NP is the same as QH06 viruses.

The phylogenetic and molecular evolutionary analyses were conducted using the neighbour-joining method with 1000 times bootstrapping implemented in the phylip 3.6 package (Felsenstein, 1989). The programs seqboot, dnadist, neighbor and consense were used. The Bayesian and maximum-parsimony approaches for tree construction, implemented in MrBayes 3 (Ronquist & Huelsenbeck, 2003) and mega3 (Kumar *et al.*, 2004), respectively, were also applied. Specifically, the GTR model with gamma-distributed rate variation across sites and a proportion of invariable sites was used in the Bayesian framework, and the default parameters (the close neighbour-interchange search method with 1000 times bootstrap) implemented in mega3 were used for maximum-parsimony approach. Phylogenetic analyses of antigen HA illustrate the evolutionary trace of H5N1 viruses related to Lake Qinghai (Fig. 1). In 2005, the QH05 outbreak revealed the multi-lineage evolution of the QH05 viruses. Along with the birds' migration, some lineages of viruses managed to land on the countries across the flyway and these caused the epidemics in the Middle East, Europe and Africa. From the phylogenetic tree, those five isolates being likely derived from a single lineage are most similar to the isolate from Croatia, 2005 (A/Cygnus olor/Croatia/1/2005), suggesting that QH06 viruses reemerged in Lake Qinghai in the spring of 2006. Isolate A/GbhGull/QH/12/06 is most likely derived from another origin, as it is not clustered with the other five QH06 isolates and is to some extent similar to A/duck/Novosibirsk/02/05. The phylogenies of the NA genes reinforced the results, as the clade of QH06 and A/Cygnus olor/Croatia/1/2005 is supported by higher bootstrap values and A/GbhGull/QH/12/06 is separated from this clade (Fig. 1).

What are the origins of the re-emerging H5N1, imported from outside or evolved locally? The phylogenetic analyses of H5N1 viruses around years 2005 and 2006 suggest that QH06 viruses most probably came from the flyway. Though the origin of isolate A/GbhGull/QH/12/06 is harder to determine, the phylogenies illustrate its origin is likely to be one coming from the flyway other than directly from QH05. Taking together that QH05 viruses have been spread out to the flyway, it seems that migratory birds have brought the variants of QH05 viruses back to Lake Qinghai as the carriers. On the other hand, the QH05 viruses might have lived in the resident birds, although we and others have failed to isolate any H5N1 virus despite a great effort after the 2005 outbreak. Therefore, we are not convinced that the possibility of local evolution should be excluded and this needs further investigation with more isolates in the future.

It is known that there are three main flyways across the Eurasia, the East Asian/Australasian flyway, the Central Asian flyway and the Black Sea/Mediterranean flyway (Fig. 2). Lake Qinghai as a congregation and breeding site is important to many migratory birds. Every March–April, birds from the south and south-west stop here for breeding, and meanwhile some local wintering populations of Lake Qinghai fly north for propagation (red arrows in Fig. 2). As a consequence, the QH05 viruses would be brought into Mongolia, Kazakhstan, Russia and Siberia through the migration along the East Asian/Australasian flyway. This is in agreement with the fact that similar viruses were isolated in Mongolia and Russia. It should be noted that the three flyways mentioned above the intersect in Siberia, so that the viruses could be spread into the Central Asian flyway and the Black Sea/Mediterranean flyway along with migratory birds flying south-west for overwintering (green arrows in Fig. 2). The outbreaks in Europe and Africa, from 2005 to 2006 are the results of the bird migration. Specifically, in the late autumn of 2005, outbreaks of H5N1 viruses were reported in Romania, Turkey, Croatia and Ukraine, respectively. Subsequently, the virus spread to the north and west with the movement of waterfowls under a blast of cold weather in January 2006 (grey arrow in Fig. 2) (Kilpatrick *et al.*, 2006), then the whole of Europe had plunged into H5N1 influenza endemic. In the beginning of 2006, the birds overwintering in the Mediterranean would fly north-east to Siberia, the flyway intersection (purple arrow in Fig. 2) for propagation. These migratory birds would probably be infected with QH05 variants. Then, some birds in the flyway intersection would migrate to central Asia, Mongolia–China and Korea–Japan. The new variants were transmitted into these areas (purple arrows in Fig. 2) and successively caused the epidemics from north to south.

Considering previous results and assumptions, we suspected that QH05 viruses have travelled through Eurasia by hitchhiking on migratory bird ‘shuttles’. They first arrived at the flyway intersection by taking Lake Qinghai-to-Russia shuttles, and then transferred to Russia-to-Mediterranean ones and reached Europe. In Europe, some are still moving south to Africa, some are travelling north-west via the waterfowl and some are boarding Africa-to-Mediterranean-to-Russia birds back to the flyway intersection. Finally, the viruses are airborne to central Asia and Korea–Japan, respectively, as well as back to Lake Qinghai. After ‘one year of travelling’, the QH05 viruses have evolved to QH06 ones as the products. Our extrapolations of the roles of the migratory birds are proposed on the basis of the phylogenetic evidence, but they still need further investigation. Nowadays, the pathogenic influenza viruses have spread beyond the Eurasian flyways and would further diffuse over more flyways. To avoid the grave consequences, more attention must be paid to the control of the AIVs, especially those HPAI virus variants, which have infected mammals and even humans (Smith *et al.*, 2006b). Besides the HPAI virus, the low pathogenic AIV may also impact the wild birds clinically, epidemiologically and ecologically (van Gils *et al.*, 2007). Therefore, in combating bird flu, surveillance should be applied to not only the domestic poultry, but also the wild birds; and multi-disciplinary research should be considered together in AIV progressive controls (FAO *et al.*, 2005).

## Figures and Tables

**Fig. 1. f1:**
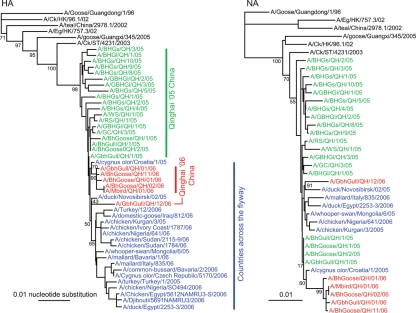
Phylogenetic analyses of HA, NA segments of H5N1 influenza viruses. All the viruses consist of QH06 viruses, QH05 viruses and isolates from countries of Asia, Europe and Africa along migratory bird flyway. The phylogenetic tree was generated using the neighbour-joining method. Analyses were based on nucleotides of full-length sequences. The bootstrap values are at branch points. QH06 viruses are labelled in red, QH05 viruses are labelled in green and isolates from countries across the flyway are labelled in blue.

**Fig. 2. f2:**
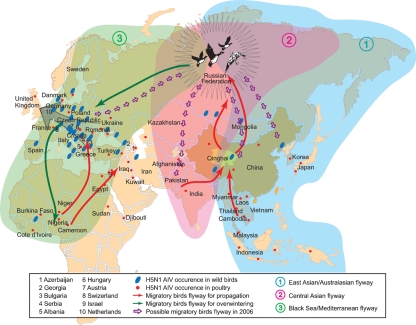
The potential routes of migratory birds from 2005 to 2006. Three flyways are shaded in green, blue and cyan, respectively. The red and blue dots illustrate the H5N1 occurrence. The arrows in red, green and purple denote the directions of the migratory birds. The coloured shadows are the ranges of different flyways. The grey arrow in Europe represents the cold weather. The cartoon birds highlight the congregation area of the wild birds from three different flyways.

**Table 1. t1:** Antigenic analyses of H5N1 influenza viruses by HI test Abbreviations: QH06, A/BhGoose/QH/01/06; QH05, A/BhGoose/QH/1/05; BJ03, A/Beijing/01/2003; VN04, A/Vietnam/1194/2004. The titres to prototype viruses are in bold.

**Virus**	**Antisera (mouse) of**			
	**QH06**	**QH05**	**BJ03**	**VN04**
QH06	**256**	256	128	32
QH05	256	**256**	128	32
BJ03	128	128	**256**	64
VN04	32	32	64	**128**
